# Use of Mechanical Circulatory Support Devices Among Patients With Acute Myocardial Infarction Complicated by Cardiogenic Shock

**DOI:** 10.1001/jamanetworkopen.2020.37748

**Published:** 2021-02-22

**Authors:** Sanket S. Dhruva, Joseph S. Ross, Bobak J. Mortazavi, Nathan C. Hurley, Harlan M. Krumholz, Jeptha P. Curtis, Alyssa P. Berkowitz, Frederick A. Masoudi, John C. Messenger, Craig S. Parzynski, Che G. Ngufor, Saket Girotra, Amit P. Amin, Nilay D. Shah, Nihar R. Desai

**Affiliations:** 1 University of California, San Francisco School of Medicine, San Francisco; 2 Section of Cardiology, Department of Medicine, San Francisco Veterans Affairs Medical Center, San Francisco, California; 3 Center for Outcomes Research and Evaluation, Yale-New Haven Hospital, New Haven, Connecticut; 4 Section of General Internal Medicine, Department of Internal Medicine, Yale School of Medicine, New Haven, Connecticut; 5 Department of Health Policy and Management, Yale School of Public Health, New Haven, Connecticut; 6 Department of Computer Science and Engineering, Texas A&M University, College Station; 7 Center for Remote Health Technologies and Systems, Texas A&M University, College Station; 8 Section of Cardiovascular Medicine, Department of Internal Medicine, Yale School of Medicine, New Haven, Connecticut; 9 Division of Cardiology, Department of Medicine, University of Colorado Anschutz Medical Campus, Aurora; 10 Robert D. and Patricia E. Kern Center for the Science of Health Care Delivery, Mayo Clinic, Rochester, Minnesota; 11 Division of Digital Health Sciences, Department of Health Sciences Research, Mayo Clinic, Rochester, Minnesota; 12 Division of Cardiovascular Diseases, Department of Internal Medicine, Carver College of Medicine, University of Iowa, Iowa City; 13 Center for Access & Delivery Research & Evaluation (CADRE), Iowa City Veterans Affairs Medical Center, Iowa City, Iowa; 14 Cardiovascular Division, Washington University School of Medicine, St Louis, Missouri; 15 Division of Health Care Policy Research, Mayo Clinic, Rochester, Minnesota

## Abstract

**Importance:**

Mechanical circulatory support (MCS) devices, including intravascular microaxial left ventricular assist devices (LVADs) and intra-aortic balloon pumps (IABPs), are used in patients who undergo percutaneous coronary intervention (PCI) for acute myocardial infarction (AMI) complicated by cardiogenic shock despite limited evidence of their clinical benefit.

**Objective:**

To examine trends in the use of MCS devices among patients who underwent PCI for AMI with cardiogenic shock, hospital-level use variation, and factors associated with use.

**Design, Setting, and Participants:**

This cross-sectional study used the CathPCI and Chest Pain-MI Registries of the American College of Cardiology National Cardiovascular Data Registry. Patients who underwent PCI for AMI complicated by cardiogenic shock between October 1, 2015, and December 31, 2017, were identified from both registries. Data were analyzed from October 2018 to August 2020.

**Exposures:**

Therapies to provide hemodynamic support were categorized as intravascular microaxial LVAD, IABP, TandemHeart, extracorporeal membrane oxygenation, LVAD, other devices, combined IABP and intravascular microaxial LVAD, combined IABP and other device (defined as TandemHeart, extracorporeal membrane oxygenation, LVAD, or another MCS device), or medical therapy only.

**Main Outcomes and Measures:**

Use of MCS devices overall and specific MCS devices, including intravascular microaxial LVAD, at both patient and hospital levels and variables associated with use.

**Results:**

Among the 28 304 patients included in the study, the mean (SD) age was 65.4 (12.6) years and 18 968 were men (67.0%). The overall MCS device use was constant from the fourth quarter of 2015 to the fourth quarter of 2017, although use of intravascular microaxial LVADs significantly increased (from 4.1% to 9.8%; *P* < .001), whereas use of IABPs significantly decreased (from 34.8% to 30.0%; *P* < .001). A significant hospital-level variation in MCS device use was found. The median (interquartile range [IQR]) proportion of patients who received MCS devices was 42% (30%-54%), and the median proportion of patients who received intravascular microaxial LVADs was 1% (0%-10%). In multivariable analyses, cardiac arrest at first medical contact or during hospitalization (odds ratio [OR], 1.82; 95% CI, 1.58-2.09) and severe left main and/or proximal left anterior descending coronary artery stenosis (OR, 1.36; 95% CI, 1.20-1.54) were patient characteristics that were associated with higher odds of receiving intravascular microaxial LVADs only compared with IABPs only.

**Conclusions and Relevance:**

This study found that, among patients who underwent PCI for AMI complicated by cardiogenic shock, overall use of MCS devices was constant, and a 2.5-fold increase in intravascular microaxial LVAD use was found along with a corresponding decrease in IABP use and a significant hospital-level variation in MCS device use. These trends were observed despite limited clinical trial evidence of improved outcomes associated with device use.

## Key Points

**Question:**

What are the trends in the use of mechanical circulatory support (MCS) devices, including intravascular microaxial left ventricular assist devices (LVADs) and intra-aortic balloon pumps, among patients who underwent percutaneous coronary intervention for acute myocardial infarction complicated by cardiogenic shock?

**Findings:**

In this cross-sectional study of 28 304 patients, the use of intravascular microaxial LVADs increased between October 2015 and December 2017, with a corresponding relative decrease in use of intra-aortic balloon pumps and significant hospital-level variation in MCS device use.

**Meaning:**

Results of this study indicated that, despite little to no evidence from clinical trials that demonstrates improved outcomes with use of MCS devices in the setting of acute myocardial infarction, persistent use of MCS devices was observed, including a substantial increase in intravascular microaxial LVADs.

## Teaser

This cross-sectional study examines the demographic, clinical, and institutional characteristics as well as other features of users of hemodynamic support therapies after a percutaneous coronary intervention for acute myocardial infarction with cardiogenic shock.

## Introduction

Intra-aortic balloon pumps (IABPs) have been the mainstay of mechanical circulatory support (MCS) for patients with cardiogenic shock in the setting of acute myocardial infarction (AMI).^1^ However, randomized clinical trial (RCT) data^2,3^ and subsequent meta-analyses^4,5^ have reported no clinical benefit from routine IABP use in patients with AMI complicated by cardiogenic shock. Impella devices (intravascular microaxial left ventricular assist devices [LVADs]), which offer greater improvement in hemodynamic parameters compared with IABPs,^6^ received US marketing clearance in 2008 for providing partial circulatory support for up to 6 hours using an extracorporeal bypass control unit and providing circulatory support during procedures not requiring cardiopulmonary bypass.^7^ Studies through 2012 showed a substantial uptake of these devices, from 4.6 per million hospital discharges in 2007 to 138 per million discharges in 2012,^8,9^ despite the absence of demonstrated benefits for hard clinical end points in RCTs.^6,10^ National Cardiovascular Data Registry (NCDR) records through September 2013 showed that use of MCS devices other than IABP was clustered around a relatively small number of hospitals but did not increase.^9^

Despite the substantial risk of death associated with cardiogenic shock^11^ and the relatively high cost of some MCS devices,^8,12^ the temporal and contemporary trends in MCS device use have not been examined in terms of detailed demographic and clinical characteristics abstracted from medical records, such as coronary anatomy. Furthermore, previous studies have focused on IABPs and other MCS devices, providing no granularity about other MCS therapies such as extracorporeal membrane oxygenation (ECMO). Understanding changes in use as well as the patients likely to receive MCS devices and the hospitals that are likely to use these devices is particularly important given the recent safety concerns about intravascular microaxial LVADs.^12,13^ In this retrospective cross-sectional study, we collected data from 2 national US registries (of the American College of Cardiology NCDR) to examine trends in the use of MCS devices, providing greater granularity of the clinical characteristics and device type than previous studies, among a large cohort of patients who underwent percutaneous coronary intervention (PCI) for AMI complicated by cardiogenic shock. We also examined hospital-level use variation and factors associated with use.

## Methods

The Human Investigation Committee of the Yale University School of Medicine approved the use of a limited data set from the NCDR for research purposes without requiring informed consent because all of the data were deidentified and maintained centrally by the NCDR. This cross-sectional study followed the Strengthening the Reporting of Observational Studies in Epidemiology (STROBE) reporting guideline.

### Data Sources and Study Population

We linked the NCDR CathPCI and Chest Pain-MI registries, both of which have been described previously.^14,15^ In brief, the CathPCI Registry is a voluntary registry of diagnostic cardiac catheterizations and PCIs performed in the US. More than 1500 hospitals across the US participate in this program and are required to submit data on all PCI procedures. The Chest Pain-MI Registry includes patients with AMI. The CathPCI Registry, version 4.4, identifies whether a patient received an IABP or any other MCS device and the timing of MCS. Version 2.4.2 of the Chest Pain-MI Registry data collection form, released in the third quarter of 2015, includes the type of MCS device.

We identified all patients who underwent PCI for AMI complicated by cardiogenic shock between October 1, 2015, and December 31, 2017, and had available data in both registries. We included individuals in the Chest Pain-MI Registry who had cardiogenic shock at first medical contact or as an in-hospital event or individuals in the CathPCI Registry who had cardiogenic shock within 24 hours prior to the PCI, at the start of the PCI, or as an intra- or postprocedure event. Cardiogenic shock was defined in both registries as systolic blood pressure less than 90 mm Hg and/or cardiac index lower than 2.2 L/min/m^2^ for at least 30 minutes that was secondary to ventricular dysfunction and/or a requirement for parenteral inotropic or vasopressor therapy or MCS devices to support blood pressure and cardiac index.^16^ For patients who underwent multiple PCIs during the hospitalization, we included data from only the initial PCI.

### Hemodynamic Support and Covariates

We categorized patients according to the hemodynamic support that they received. The CathPCI Registry details if a patient received an IABP or a different MCS device. The Chest Pain-MI Registry details if a patient received an IABP, intravascular microaxial LVAD, TandemHeart (CardiacAssist Inc), ECMO, LVAD, or other device. The Chest Pain-MI Registry allows documentation of only 1 MCS device per patient. Therefore, by linking the 2 registries, we could identify the MCS devices used (Chest Pain-MI Registry) in combination with IABPs (CathPCI Registry). Patients who did not receive any MCS device composed the medical therapy only group.

Patient-level covariates were patient demographic characteristics, medical history, and clinical presentation. Hospital-level covariates were number of beds, location, type (government, private, or university), presence of teaching program, and mean annual PCI volume. For continuous values with missing values, the mean was imputed. For binary (yes or no) variables, all missing variables were coded as no; for categorical variables, all missing variables were coded as no or other (if a no category did not exist).

### Statistical Analysis

We characterized overall MCS device use, including for specific sociodemographic and clinical subgroups (age, sex, race, insurance status, ST-segment elevation MI [STEMI] or non-STEMI, cardiac arrest or no arrest, and transfer status). We examined trends in the use of hemodynamic support by calendar quarter using the Cochrane-Armitage test to determine the significance of changes over time.

We performed multivariable logistic regression to identify independent variables associated with MCS device use compared with medical therapy among all patients with AMI complicated by cardiogenic shock, accounting for clustering by facility (ie, accounting for the possible associations among patients who received care at a given hospital such that the observations were not independent). The model included demographic variables (age, sex, race, and insurance status), comorbidities (previous PCI, previous coronary artery bypass graft [CABG], and peripheral artery disease), clinical presentation variables (cardiac arrest at first medical contact or during hospitalization, STEMI, anterior infarction, left main or proximal left anterior descending coronary artery [LAD] disease, and left ventricular ejection fraction), and hospital variables (number of beds, location, type, teaching program, and mean annual PCI volume). Using the same model, we performed an additional multivariable logistic regression to examine the odds of a patient receiving an intravascular microaxial LVAD compared with an IABP, restricting the analyses to patients with AMI complicated by cardiogenic shock who received either an IABP or intravascular microaxial LVAD only.

We examined hospital-level variation in MCS device use among hospitals that cared for at least 10 patients with AMI complicated by cardiogenic shock during the study period. We calculated a median odds ratio (OR) by building a generalized linear mixed model with random hospital intercepts. The median OR (always ≥1) was derived from the estimate of the variance of the random intercept of the model.^17^ Conceptually, the median OR represents the relative odds for 2 identical patients receiving an MCS device at 1 randomly selected hospital vs another randomly selected hospital. A median OR of 1.0 indicates no hospital-level variation, whereas a median OR of 2 indicates that the odds of receiving an MCS device are 2-fold higher in 1 randomly selected hospital vs another hospital. Using the same methods, we calculated a hospital-specific median OR for a patient with AMI complicated by cardiogenic shock to receive an intravascular microaxial LVAD.

We compared hospital characteristics (number of beds, location, type, teaching program, and mean annual PCI volume) by quartiles of MCS device use. We also compared the characteristics of hospitals that used at least 1 intravascular microaxial LVAD vs hospitals that did not. Among hospitals that used at least 1 intravascular microaxial LVAD, we compared the characteristics by tertiles. We used χ^2^ test for categorical variables and Kruskal-Wallis test for non-normally distributed continuous variables.

All statistical analyses were 2-sided, with an α = .05 for statistical significance. All analyses were conducted in R, version 3.6.0 (R Foundation for Statistical Computing), with packages clubSandwich 0.4.2^18^; ggplot2, version 3.2.1^19^; DescTools 0.99.34^20^; and lubridate 1.7.4.^21^ Data were analyzed from October 2018 to August 2020.

## Results

### MCS Device Use and Change Over Time

Among 28 304 patients with AMI complicated by cardiogenic shock who received PCI at 928 hospitals during the study period, the mean (SD) age was 65.4 (12.6) years and 18 968 were men (67.0%). Overall, 12 077 patients (42.7%) received an MCS device and 16 227 (57.3%) received medical therapy only during the hospitalization. Of the 12 077 patients who received an MCS device, 1768 (14.6%) received an intravascular microaxial LVAD only, 8471 (70.1%) received an IABP only, 5 (0%) received TandemHeart, 182 (1.5%) received ECMO, 23 (0.2%) received an LVAD, 276 (2.3%) received both an IABP and intravascular microaxial LVAD, 4 (0%) received an IABP and TandemHeart, 138 (1.1%) received an IABP and ECMO, 17 (0.1%) received an IABP and LVAD, and 1193 (9.9%) received another MCS device or a combination of MCS devices (eFigure 1 in the Supplement).

**Figure 1.**
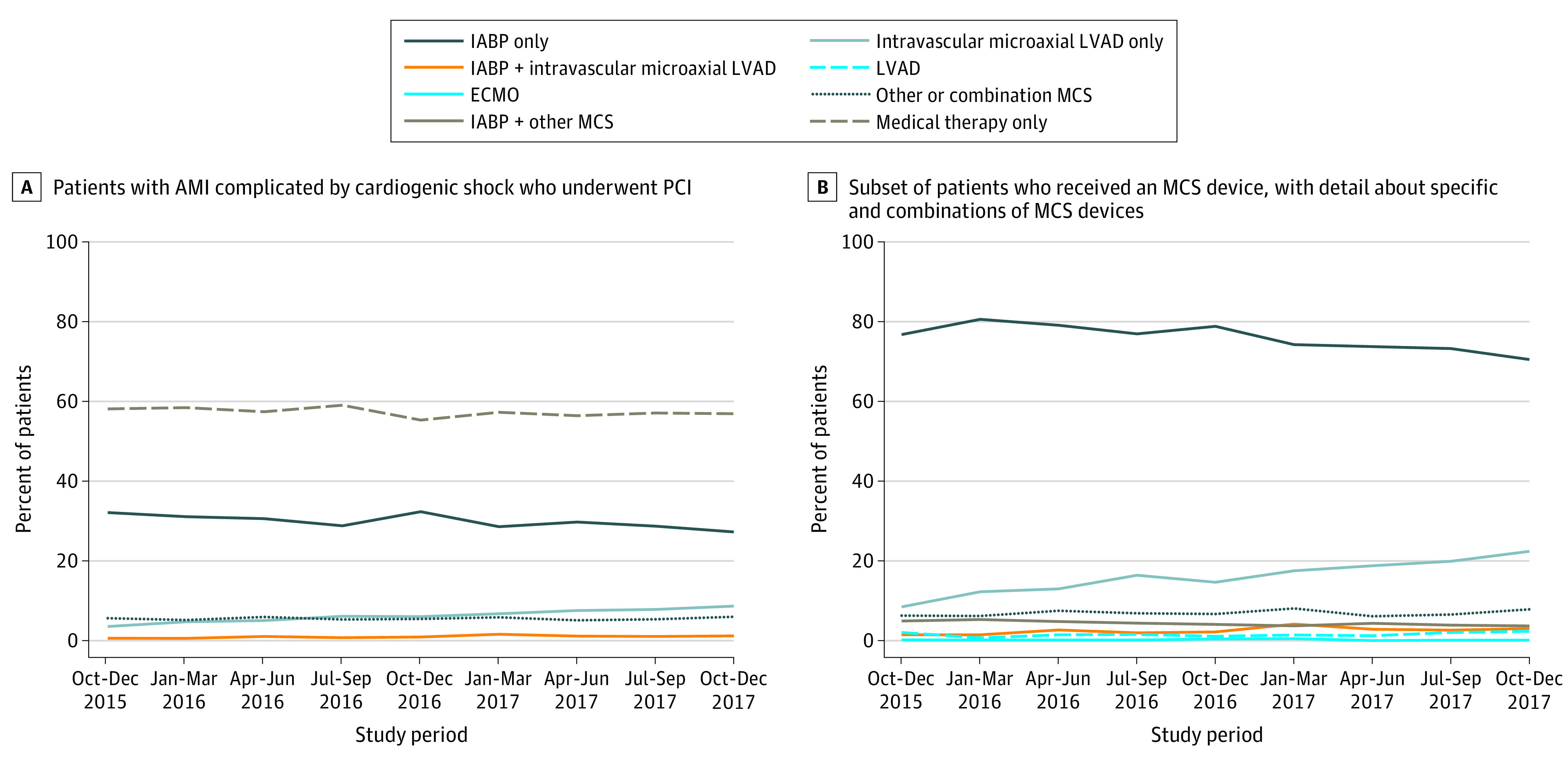
Quarterly Use of Mechanical Circulatory Support (MCS) Devices for Patients Who Underwent Percutaneous Coronary Intervention (PCI) for Acute Myocardial Infarction (AMI) Complicated by Cardiogenic Shock From October 2015 to December 2017 at Hospitals Participating in the National Cardiovascular Data Registry CathPCI and Chest Pain-MI Registries Patients were categorized according to those receiving an intra-aortic balloon pump (IABP) only, intravascular microaxial left ventricular assist device (LVAD) only, IABP and intravascular microaxial LVAD, LVAD, extracorporeal membrane oxygenation (ECMO), IABP and other MCS devices, and other MCS devices or combination of MCS devices.

During the study period, the proportion of patients who used any MCS device remained similar from October through December 2015 to October through December 2017 (from 41.9% to 43.1%; *P* = .07) (Figure 1A). A significant increase in the use of intravascular microaxial LVADs (either alone or in combination with IABPs) was found (from 4.1% to 9.8%; *P* < .001) during this period along with a corresponding decrease in the percentage of patients who received IABPs either alone or in combination with other MCS devices (from 34.8% to 30.0%; *P* < .001). When limited to patients receiving any MCS, the use of intravascular microaxial LVADs increased from 9.9% to 20.6%, whereas IABP use decreased from 83.1% to 73.2% (Figure 1B).

### Hospital-Level Variation in MCS Device Use

**Figure 2.**
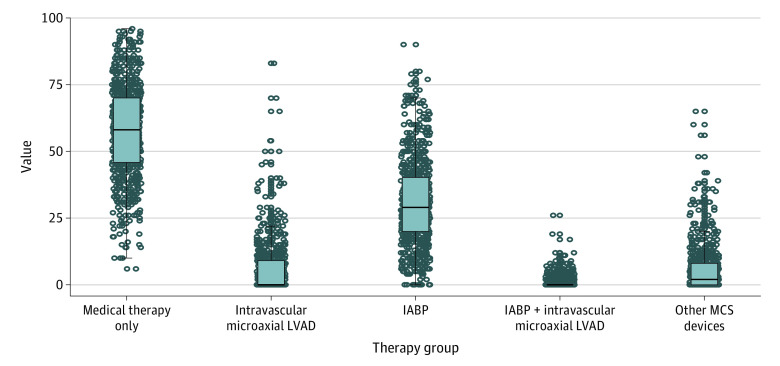
Proportion of Hospitals That Used Mechanical Circulatory Support (MCS) Devices for Patients Who Underwent Percutaneous Coronary Intervention for Acute Myocardial Infarction Complicated by Cardiogenic Shock From October 2015 to December 2017 The horizontal lines in the center of each box indicate the median; the lower and upper bounds of each box, the 25th and 75th percentiles; and error bars, 1.5 times the interquartile range. Each hospital is represented as a point. Only hospitals with at least 10 cases of cardiogenic shock during the study period were included. IABP indicates intra-aortic balloon pump; LVAD, left ventricular assist device.

Of the 928 hospitals included in the study, 521 (56.1%) did not use any intravascular microaxial LVADs for patients with AMI complicated by cardiogenic shock. Among hospitals with at least 10 cases of AMI with cardiogenic shock during the study period, a significant variation in MCS device use was observed (Figure 2). The median (interquartile range [IQR]) proportion of patients who received an MCS device at the hospital level was 42% (30%-54%; range 4%-94%). The median (IQR) proportion of patients who received any intravascular microaxial LVAD was 1% (0%-10%; range, 0%-83%).

The hospital-specific median OR for use of any MCS device over the study period was 1.79 (95% CI, 1.71-1.86). This OR indicates that the odds of receiving an MCS device were 1.79-fold higher in 1 randomly selected hospital vs another. The hospital-specific median OR for use of any intravascular microaxial LVAD only over the study period was 3.33 (95% CI, 3.03-3.63). This OR indicates that the odds of receiving an intravascular microaxial LVAD were 3.33-fold higher in 1 randomly selected hospital vs another.

### MCS Device Use by Hospital Characteristics

Among all hospitals that cared for patients with AMI complicated by cardiogenic shock, larger hospitals (≥600 beds) were more likely to be in higher quartiles of MCS device use and smaller ones (≤200 beds) were more likely to be in the lowest quartile of MCS device use (Table 1). University hospitals and those with teaching programs were more likely to be in higher quartiles of MCS device use. Hospitals with higher mean annual PCI volumes were more likely to use MCS devices. No significant difference in MCS device use was found across hospitals that were rural, suburban, or urban.

**Table 1.** Hospital Characteristics After Stratification by Quartiles of Use of Any Mechanical Circulatory Support (MCS) Device

Characteristic	Any use of MCS device				
	Quartile 1 (n = 230)	Quartile 2 (n = 233)	Quartile 3 (n = 229)	Quartile 4 (n = 236)	*P* value
Patients with AMI complicated by cardiogenic shock who underwent PCI and received an MCS device at each hospital, %	<29	29 to <42	≥42 to <55	≥55	NA
Beds, No. (%)					.005
<200	77 (33.5)	65 (27.9)	47 (20.5)	60 (25.4)	
200-399	100 (43.5)	96 (41.2)	91 (39.7)	93 (39.4)	
400-599	39 (17.0)	37 (15.9)	51 (22.3)	47 (19.9)	
≥600	14 (6.1)	35 (15.0)	40 (17.5)	36 (15.3)	
Location					.96
Rural	45 (19.6)	40 (17.2)	36 (15.7)	44 (18.6)	
Suburban	79 (34.3)	80 (34.3)	79 (34.5)	81 (34.3)	
Urban	106 (46.1)	113 (48.5)	114 (49.8)	111 (47.0)	
Type					<.001
Government	7 (3.0)	4 (1.7)	3 (1.3)	1 (0.4)	
Private	215 (93.5)	216 (92.7)	195 (85.2)	205 (86.9)	
University	8 (3.5)	13 (5.6)	31 (13.5)	30 (12.7)	
Teaching program	68 (29.6)	92 (39.5)	104 (45.4)	106 (44.9)	.001
Annual PCI volume, mean (SD)	482.9 (521.4)	546.0 (458.5)	681.0 (644.6)	584.3 (553.4)	<.001

Abbreviations: AMI, acute myocardial infarction; NA, not applicable; PCI, percutaneous coronary intervention.

Hospitals that placed at least 1 intravascular microaxial LVAD for patients who underwent PCI for AMI complicated by cardiogenic shock were more likely to be large (≥200 beds), be in an urban setting, have a teaching program, and have a higher annual PCI volume (Table 2). Across tertiles of hospitals that used intravascular microaxial LVADs, no significant difference was observed in the number of beds, location, type, or presence of teaching program. Hospitals with lower annual PCI volume were more likely to be in the highest tertile of intravascular microaxial LVAD use.

**Table 2.** Hospital Characteristics After Stratification by Use of Intravascular Microaxial Left Ventricular Assist Device (LVAD)

Characteristic	No use of intravascular microaxial LVAD (n = 521)	Any (at least 1) use of intravascular microaxial LVAD				*P* value (no vs any use)
		Tertile 1 (n = 125)	Tertile 2 (n = 135)	Tertile 3 (n = 147)	*P* value (among tertiles)	
Patients with AMI complicated by cardiogenic shock who underwent PCI and received an intravascular microaxial LVAD at each hospital, %	NA	<7	7 to <15	≥15	NA	NA
Beds, No. (%)					.50	<.001
<200	179 (34.4)	16 (12.8)	23 (17.0)	31 (21.1)		
200-399	205 (39.3)	54 (43.2)	59 (43.7)	62 (42.2)		
400-599	89 (17.1)	30 (24.0)	24 (17.8)	31 (21.1)		
≥600	48 (9.2)	25 (20.0)	29 (21.5)	23 (15.6)		
Location					.83	.003
Rural	105 (20.2)	18 (14.4)	21 (15.6)	21 (14.3)		
Suburban	192 (36.9)	38 (30.4)	38 (28.1)	51 (34.7)		
Urban	224 (43.0)	69 (55.2)	76 (56.3)	75 (51.0)		
Type					.76	.17
Government	9 (1.7)	1 (0.8)	3 (2.2)	2 (1.4)		
Private	474 (91.0)	108 (86.4)	120 (88.9)	129 (87.8)		
University	38 (7.3)	16 (12.8)	12 (8.9)	16 (10.9)		
Teaching program	184 (35.3)	57 (45.6)	69 (51.1)	60 (40.8)	.22	.001
Annual PCI volume, mean (SD)	442.1 (498.1)	821.3 (562.7)	753.4 (666.7)	662.8 (471.2)	.03	<.001

Abbreviations: AMI, acute myocardial infarction; NA, not applicable; PCI, percutaneous coronary intervention.

### MCS Device Use by Patient Demographic and Clinical Characteristics

In comparing the unadjusted use of intravascular microaxial LVADs only with use of IABPs only within a denominator of all therapies for AMI complicated by cardiogenic shock, men were more likely than women to receive intravascular microaxial LVADs (6.6% vs 5.4%; *P* < .01) and IABPs (30.9% vs 27.9%; *P* < .01) (eFigure 2 in the Supplement). Patients younger than 65 years were more likely to receive intravascular microaxial LVADs than those aged 75 years or older (6.5% vs 5.4%; *P* = .002) (eFigure 3 in the Supplement). Black patients were significantly more likely to receive intravascular microaxial LVADs compared with patients who were not Black individuals (7.5% vs 6.5%; *P* = .005) (eFigure 4 in the Supplement). Additional analyses that characterize the use of MCS devices by insurance, type of myocardial infarction, cardiac arrest status, and transfer status are provided in eFigures 5 to 8 in the Supplement.

### Characteristics Associated With MCS Device Use and With Intravascular Microaxial LVAD vs IABP Use

In multivariable regression analysis, female sex (OR, 0.88; 95% CI, 0.83-0.93), the presence of peripheral artery disease (OR, 0.78; 95% CI, 0.71-0.86), and previous CABG (OR, 0.74; 95% CI, 0.67-0.81) were associated with lower odds of receiving any MCS device (Table 3). Private insurance (vs no insurance), cardiac arrest at first medical contact or during hospitalization, STEMI, anterior infarction, and severe left main and/or proximal LAD stenosis were associated with greater MCS device use. Patients treated at private or university hospitals were more likely to receive any MCS device.

**Table 3.** Patient and Hospital Characteristics Associated With Use of Any Mechanical Circulatory Support (MCS) Device vs Medical Therapy Only and With Use of Intravascular Microaxial Left Ventricular Assist Device (LVAD) Only vs Intra-Aortic Balloon Pump Only

Variable	OR (95% CI)	
	Use of any MCS drvice vs medical therapy only	Use of intravascular microaxial LVAD vs intra-aortic balloon pump
**Patient characteristics**		
Age	1.00 (1.00-1.00)	1.00 (0.99-1.00)
Female sex	0.88 (0.83-0.93)	0.93 (0.82-1.05)
BMI	1.01 (1.00-1.01)	1.02 (1.01-1.03)
Race		
Other^a^	1 [Reference]	1 [Reference]
White	0.88 (0.77-1.00)	1.04 (0.75-1.46)
Black	0.86 (0.74-1.00)	1.29 (0.86-1.94)
Insurance		
None	1 [Reference]	1 [Reference]
Medicaid	1.06 (0.92-1.22)	0.87 (0.64-1.16)
Medicare	1.11 (0.99-1.24)	0.93 (0.73-1.18)
Private	1.13 (1.03-1.23)	1.00 (0.81-1.24)
Medicaid and Medicare	0.97 (0.84-1.13)	0.91 (0.65-1.28)
Private and public	1.06 (0.94-1.18)	0.94 (0.74-1.20)
Other or combined with other insurance	1.07 (0.93-1.24)	1.05 (0.79-1.39)
Medical history		
PAD	0.78 (0.71-0.86)	1.26 (1.05-1.52)
Cardiac arrest at first medical contact or during hospitalization	1.70 (1.58-1.83)	1.82 (1.58-2.09)
STEMI	1.19 (1.11-1.28)	0.69 (0.60-0.80)
Anterior infarction	1.19 (1.12-1.27)	0.94 (0.82-1.07)
Left main or proximal LAD disease	2.21 (2.08-2.35)	1.36 (1.20-1.54)
Previous PCI	1.00 (0.94-1.07)	1.04 (0.92-1.18)
Previous CABG	0.74 (0.67-0.81)	0.79 (0.64-0.99)
LVEF (per 1% increase)	0.96 (0.96-0.97)	0.97 (0.97-0.98)
**Hospital characteristics**		
No. of beds		
<200	1 [Reference]	1 [Reference]
200-399	1.09 (0.94-1.27)	1.04 (0.65-1.65)
400-599	1.13 (0.94-1.35)	0.98 (0.61-1.57)
≥600	1.22 (0.99-1.50)	0.81 (0.48-1.39)
Location		
Rural	1 [Reference]	1 [Reference]
Suburban	0.91 (0.77-1.08)	0.86 (0.55-1.37)
Urban	0.89 (0.75-1.05)	1.08 (0.70-1.66)
Type		
Government	1 [Reference]	1 [Reference]
Private	1.33 (0.80-2.21)	0.91 (0.44-1.85)
University	1.84 (1.08-3.12)	0.82 (0.36-1.90)
Teaching program	1.02 (0.91-1.15)	1.05 (0.79-1.39)
Mean annual PCI volume (per increase of 1 annual PCI)	1.00 (1.00-1.00)	1.00 (1.00-1.00)

Abbreviations: BMI, body mass index (calculated as weight in kilograms divided by height in meters squared); CABG, coronary artery bypass graft; LAD, left anterior descending coronary artery; LVEF, left ventricular ejection fraction; OR, odds ratio; PAD, peripheral artery disease; PCI, percutaneous coronary intervention; STEMI, ST-segment elevation myocardial infarction.

^a^ Other race included Asian, American Indian, and Native Hawaiian/Pacific Islander.

In multivariable regression analysis, patients who presented with STEMI (OR, 0.69; 95% CI, 0.60-0.80) and with previous CABG (OR, 0.79; 95% CI, 0.64-0.99) had significantly lower odds of use of intravascular microaxial LVADs only vs use of IABPs only (Table 3). Cardiac arrest at first medical contact or during hospitalization (OR, 1.82; 95% CI, 1.58-2.09) and severe left main and/or proximal LAD stenosis (OR, 1.36; 95% CI, 1.20-1.54) were associated with higher odds of intravascular microaxial LVAD use compared with IABP use.

## Discussion

This large, national cross-sectional study of patients who underwent PCI for AMI complicated by cardiogenic shock showed that, although overall use of MCS devices remained constant between 2015 and 2017, use of intravascular microaxial LVADs increased substantially, whereas use of IABPs decreased. Significant hospital-level variation in MCS device use was observed, with some hospitals not using any MCS devices and some hospitals using only intravascular microaxial LVADs or only IABPs. Other MCS devices remained infrequently used but may be used in combination with or as part of sequential therapy.

Previous studies through 2013 demonstrated a decrease in IABP use,^9^ which may be attributed to RCTs not demonstrating the clinical benefits of this device.^2,3^ This study extends these past findings to more recent years. Regardless, we found that IABP remains the most commonly used MCS device in patients with AMI complicated by cardiogenic shock; more than 70% of patients who received an MCS device received an IABP. The ongoing use of this device despite its lack of association with improved clinical outcomes may be explained by familiarity with IABPs and because clinical practice guidelines in the US have not recommended against routine IABP use.^1^ This finding is in contrast to the European Society of Cardiology clinical practice guidelines published in August 2017 (near the end of the study period), which gave routine IABP use a class III recommendation for patients with cardiogenic shock and STEMI.^22^

The increasing use of intravascular microaxial LVADs may be associated with the greater hemodynamic support they provide compared with IABPs to patients with cardiogenic shock,^6^ who have a high mortality risk. Patients expected to have greater hemodynamic compromise, including those with cardiac arrest and left main or proximal LAD disease, were more likely to receive intravascular microaxial LVADs. Some groups have recommended intravascular microaxial LVADs for patients with severe cardiogenic shock.^23^

However, the significant hospital-level variation in MCS device use and intravascular microaxial LVAD use suggests that no standard of care exists. This lack of consensus is consistent with multiple other studies, including a study of patients with AMI complicated by cardiogenic shock that reported that patient characteristics were not associated with MCS device use^24^ and with another study of patients with cardiogenic shock in cardiac intensive care units in which hospital-level variation in MCS device use could not be explained by differences in illness severity.^25^

One reason for the substantial variation in hospital use of MCS devices may be the paucity of clinical study data demonstrating the clinical benefit of intravascular microaxial LVAD use among patients with AMI complicated by cardiogenic shock.^26^ Existing RCTs do not show the benefits of IABP use in AMI with cardiogenic shock,^6,10^ although recent large observational studies have found that intravascular microaxial LVAD use was associated with higher mortality compared with IABP use.^12,13^ Intravascular microaxial LVADs were also significantly more expensive than IABPs, suggesting significant differences in total cost.^8,12,27^ Another reason for the variation in hospital-level device use could be that patient and device selection for AMI with cardiogenic shock remains uncertain because of the clinical heterogeneity of cardiogenic shock.^11^ A recently released classification scheme^28^ could help establish the specific cardiogenic shock stages under which different MCS devices should be deployed. A third reason for the hospital-level use variation may be that hospitals that have invested in the infrastructure to deploy intravascular microaxial LVADs for the care of patients are more likely to use these devices. Differences in reimbursement for intravascular microaxial LVADs vs IABPs^8^ as well as other factors may also be associated with the observed use trends. Additional RCT evidence, which would help guide the selection, use, and timing of MCS devices in patients with AMI complicated by cardiogenic shock, could play a role in reducing hospital-level variation and improving patient outcomes as well as targeting these devices to patients who are most likely to find them beneficial.^26,29^

Among patients with STEMI, we found increased odds of MCS device use but lower use of intravascular microaxial LVADs. Because patients with STEMI in general have more acute, unstable presentations and are more likely to have a cardiac arrest, it is not surprising that these patients often received MCS devices. However, in the model adjusted for clinical presentation and coronary anatomy, the lower odds of intrasvascular microaxial LVAD vs IABP use broadly highlighted the substantial variation in use trends that seemed to be associated not only with clinical presentation or physiological features but also with discretionary decision-making by physicians and institutions.

A novel finding of this study was that women with AMI complicated by cardiogenic shock were less likely than men to receive any MCS therapy. This finding extends the reports of differences in treatment provided to women with AMI, such as primary PCI^30^ and other device-based therapy for cardiovascular disease.^31^ These differences may be associated with the smaller vascular anatomy, which cannot accommodate the large bore access needed for MCS devices, and a greater predisposition to bleeding complications in women compared with men.^32^ Further research is needed to ascertain the reasons for these sex-based differences.

### Limitations

This study has several limitations. First, the presence of cardiogenic shock was based on site documentation. Second, different types of intravascular microaxial LVADs, specifically the Impella 2.5, CP, 5.0, and RP devices (ABIOMED), could not be distinguished. Third, because the Chest Pain-MI Registry allows only a single MCS device to be coded, some patients may have received combinations of devices that were not captured. Fourth, we did not have information on all variables relevant to cardiogenic shock (eg, lactate levels or number of vasopressors used), which may be associated with use of specific MCS devices.

## Conclusions

Among patients who underwent PCI for AMI complicated by cardiogenic shock from October 2015 to December 2017, use of intravascular microaxial LVADs increased, with a corresponding decrease in use of IABPs despite limited clinical trial evidence of improved outcomes associated with device use. Significant hospital-level variation in use of MCS devices was also found.

## Notes

Supplement. **eFigure 1.** Flow Diagram of Patients Undergoing Percutaneous Coronary Intervention for Acute Myocardial Infarction Complicated by Cardiogenic Shock **eFigure 2.** Sex Distribution by Therapy of Patients Undergoing Percutaneous Coronary Intervention for Acute Myocardial Infarction Complicated by Cardiogenic Shock from October 1, 2015 – December 31, 2017 **eFigure 3.** Age Distribution by Therapy for Patients Undergoing Percutaneous Coronary Intervention for Acute Myocardial Infarction Complicated by Cardiogenic Shock from October 1, 2015 – December 31, 2017 **eFigure 4.** Race Distribution by Therapy for Patients Undergoing Percutaneous Coronary Intervention for Acute Myocardial Infarction Complicated by Cardiogenic Shock from October 1, 2015 – December 31, 2017 **eFigure 5.** Insurance Distribution by Therapy for Patients Undergoing Percutaneous Coronary Intervention for Acute Myocardial Infarction Complicated by Cardiogenic Shock from October 1, 2015 – December 31, 2017 **eFigure 6.** Type of Myocardial Infarction by Therapy for Patients Undergoing Percutaneous Coronary Intervention for Acute Myocardial Infarction Complicated by Cardiogenic Shock from October 1, 2015 – December 31, 2017 **eFigure 7.** Cardiac Arrest Status by Therapy for Patients Undergoing Percutaneous Coronary Intervention for Acute Myocardial Infarction Complicated by Cardiogenic Shock from October 1, 2015 – December 31, 2017 **eFigure 8.** Therapies for Transfer Patients Undergoing Percutaneous Coronary Intervention for Acute Myocardial Infarction Complicated by Cardiogenic Shock from October 1, 2015 – December 31, 2017
