# Supplementary material for: Use of Mechanical Circulatory Support Devices Among Patients With Acute Myocardial Infarction Complicated by Cardiogenic Shock
Source: JAMA Netw Open. 2021 Feb 22;4(2):e2037748. doi: 10.1001/jamanetworkopen.2020.37748 (PMC7900859; doi:10.1001/jamanetworkopen.2020.37748)
Supplement: Supplement. — eFigure 1. Flow Diagram of Patients Undergoing Percutaneous Coronary Intervention for Acute Myocardial Infarction Complicated by Cardiogenic Shock eFigure 2. Sex Distribution by Therapy of Patients Undergoing Percutaneous Coronary Intervention for Acute Myocardial Infarction Complicated by Cardiogenic Shock from October 1, 2015 – December 31, 2017 eFigure 3. Age Distribution by Therapy for Patients Undergoing Percutaneous Coronary Intervention for Acute Myocardial Infarction Complicated by Cardiogenic Shock from October 1, 2015 – December 31, 2017 eFigure 4. Race Distribution by Therapy for Patients Undergoing Percutaneous Coronary Intervention for Acute Myocardial Infarction Complicated by Cardiogenic Shock from October 1, 2015 – December 31, 2017 eFigure 5. Insurance Distribution by Therapy for Patients Undergoing Percutaneous Coronary Intervention for Acute Myocardial Infarction Complicated by Cardiogenic Shock from October 1, 2015 – December 31, 2017 eFigure 6. Type of Myocardial Infarction by Therapy for Patients Undergoing Percutaneous Coronary Intervention for Acute Myocardial Infarction Complicated by Cardiogenic Shock from October 1, 2015 – December 31, 2017 eFigure 7. Cardiac Arrest Status by Therapy for Patients Undergoing Percutaneous Coronary Intervention for Acute Myocardial Infarction Complicated by Cardiogenic Shock from October 1, 2015 – December 31, 2017 eFigure 8. Therapies for Transfer Patients Undergoing Percutaneous Coronary Intervention for Acute Myocardial Infarction Complicated by Cardiogenic Shock from October 1, 2015 – December 31, 2017 [file jamanetwopen-e2037748-s001.pdf]

Supplementary Online Content

Dhruva SS, Ross JS, Mortazavi BJ, et al. Use of mechanical circulatory support devices among patients with acute myocardial infarction complicated by cardiogenic shock. *JAMA Netw Open*. 2021;4(2):e2037748. doi:10.1001/jamanetworkopen.2020.37748

eFigure 1. Flow Diagram of Patients Undergoing Percutaneous Coronary Intervention for Acute Myocardial Infarction Complicated by Cardiogenic Shock

eFigure 2. Sex Distribution by Therapy of Patients Undergoing Percutaneous Coronary Intervention for Acute Myocardial Infarction Complicated by Cardiogenic Shock from October 1, 2015 – December 31, 2017

eFigure 3. Age Distribution by Therapy for Patients Undergoing Percutaneous Coronary Intervention for Acute Myocardial Infarction Complicated by Cardiogenic Shock from October 1, 2015 – December 31, 2017

eFigure 4. Race Distribution by Therapy for Patients Undergoing Percutaneous Coronary Intervention for Acute Myocardial Infarction Complicated by Cardiogenic Shock from October 1, 2015 – December 31, 2017

eFigure 5. Insurance Distribution by Therapy for Patients Undergoing Percutaneous Coronary Intervention for Acute Myocardial Infarction Complicated by Cardiogenic Shock from October 1, 2015 – December 31, 2017

eFigure 6. Type of Myocardial Infarction by Therapy for Patients Undergoing Percutaneous Coronary Intervention for Acute Myocardial Infarction Complicated by Cardiogenic Shock from October 1, 2015 – December 31, 2017

eFigure 7. Cardiac Arrest Status by Therapy for Patients Undergoing Percutaneous Coronary Intervention for Acute Myocardial Infarction Complicated by Cardiogenic Shock from October 1, 2015 – December 31, 2017

eFigure 8. Therapies for Transfer Patients Undergoing Percutaneous Coronary Intervention for Acute Myocardial Infarction Complicated by Cardiogenic Shock from October 1, 2015 – December 31, 2017

This supplementary material has been provided by the authors to give readers additional information about their work.

©2021 Dhruva SS et al. *JAMA Network Open.*

Abbreviations:

AMI- acute myocardial infarction

ECMO- extracorporeal membrane oxygenation

IABP- intra-aortic balloon pump

LVAD- left ventricular assist device

MCS- mechanical circulatory support

NCDR®- National Cardiovascular Data Registry

©2021 Dhruva SS et al. *JAMA Network Open.*

eFigure 1. Flow Diagram of Patients Undergoing Percutaneous Coronary Intervention for Acute Myocardial Infarction Complicated by Cardiogenic Shock

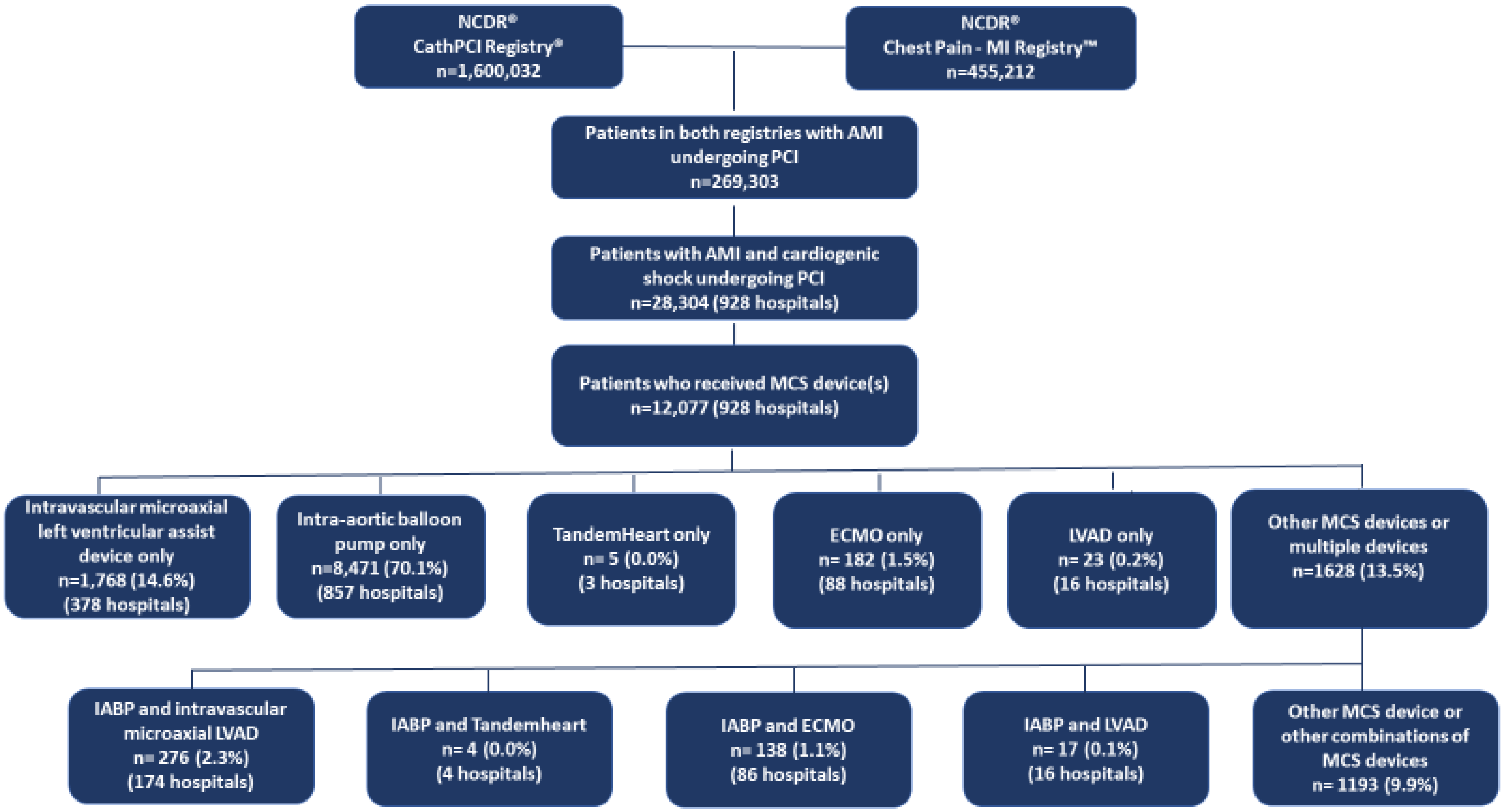

©2021 Dhruva SS et al. *JAMA Network Open.*

eFigure 2. Sex Distribution by Therapy of Patients Undergoing Percutaneous Coronary Intervention for Acute Myocardial Infarction Complicated by Cardiogenic Shock from October 1, 2015 – December 31, 2017

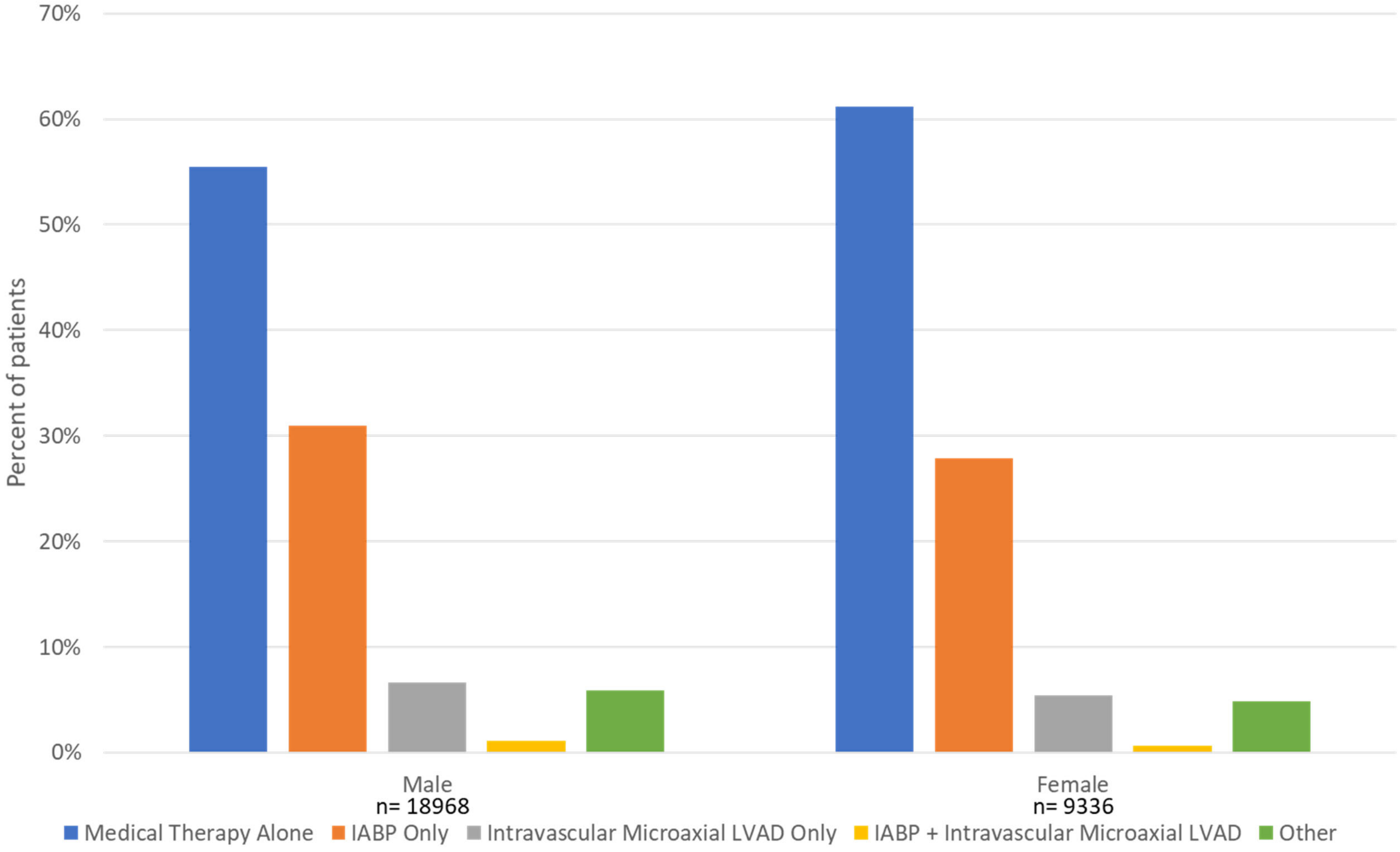

©2021 Dhruva SS et al. *JAMA Network Open.*

eFigure 3. Age Distribution by Therapy for Patients Undergoing Percutaneous Coronary Intervention for Acute Myocardial Infarction Complicated by Cardiogenic Shock from October 1, 2015 – December 31, 2017

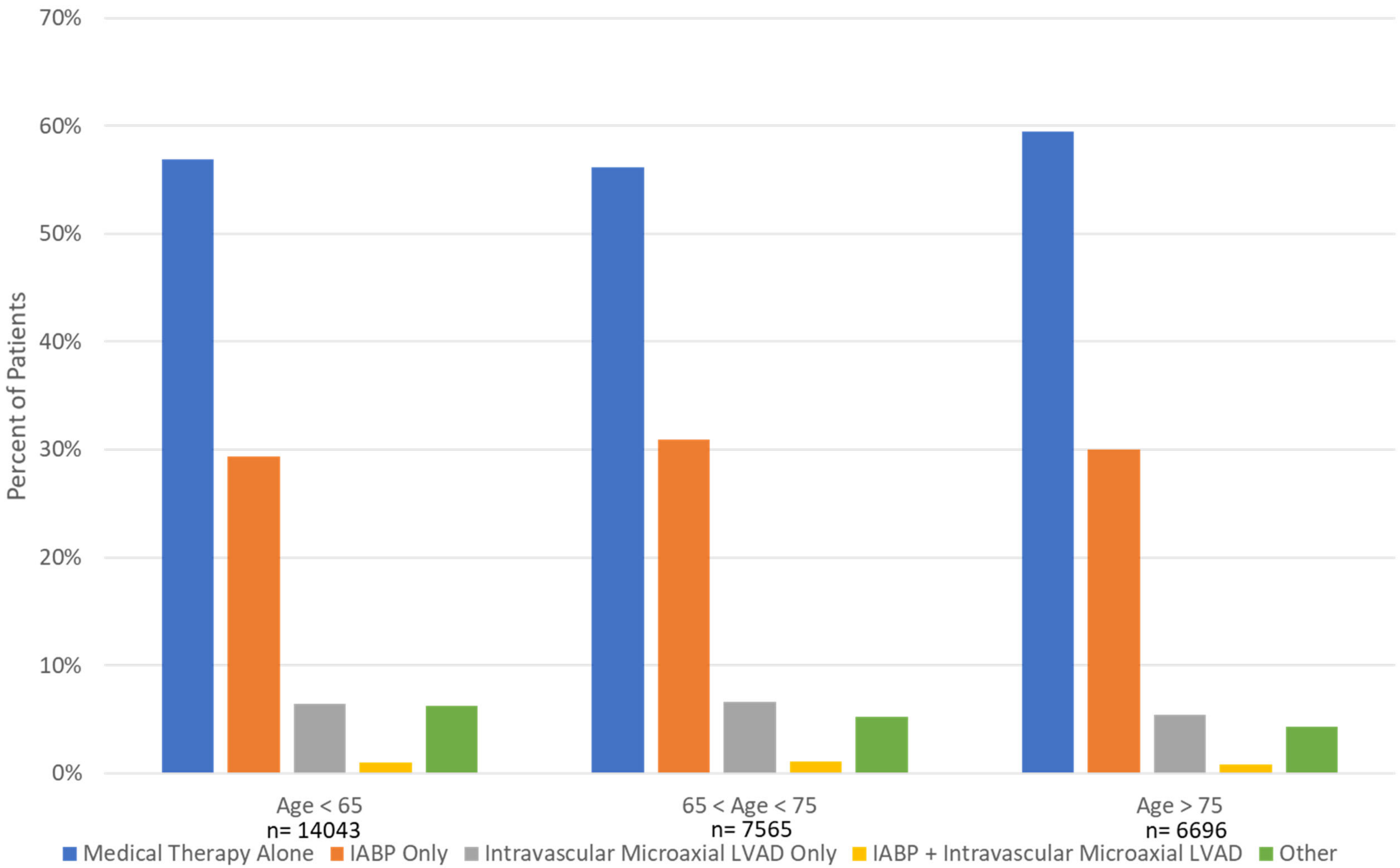

©2021 Dhruva SS et al. *JAMA Network Open.*

eFigure 4. Race Distribution by Therapy for Patients Undergoing Percutaneous Coronary Intervention for Acute Myocardial Infarction Complicated by Cardiogenic Shock from October 1, 2015 – December 31, 2017

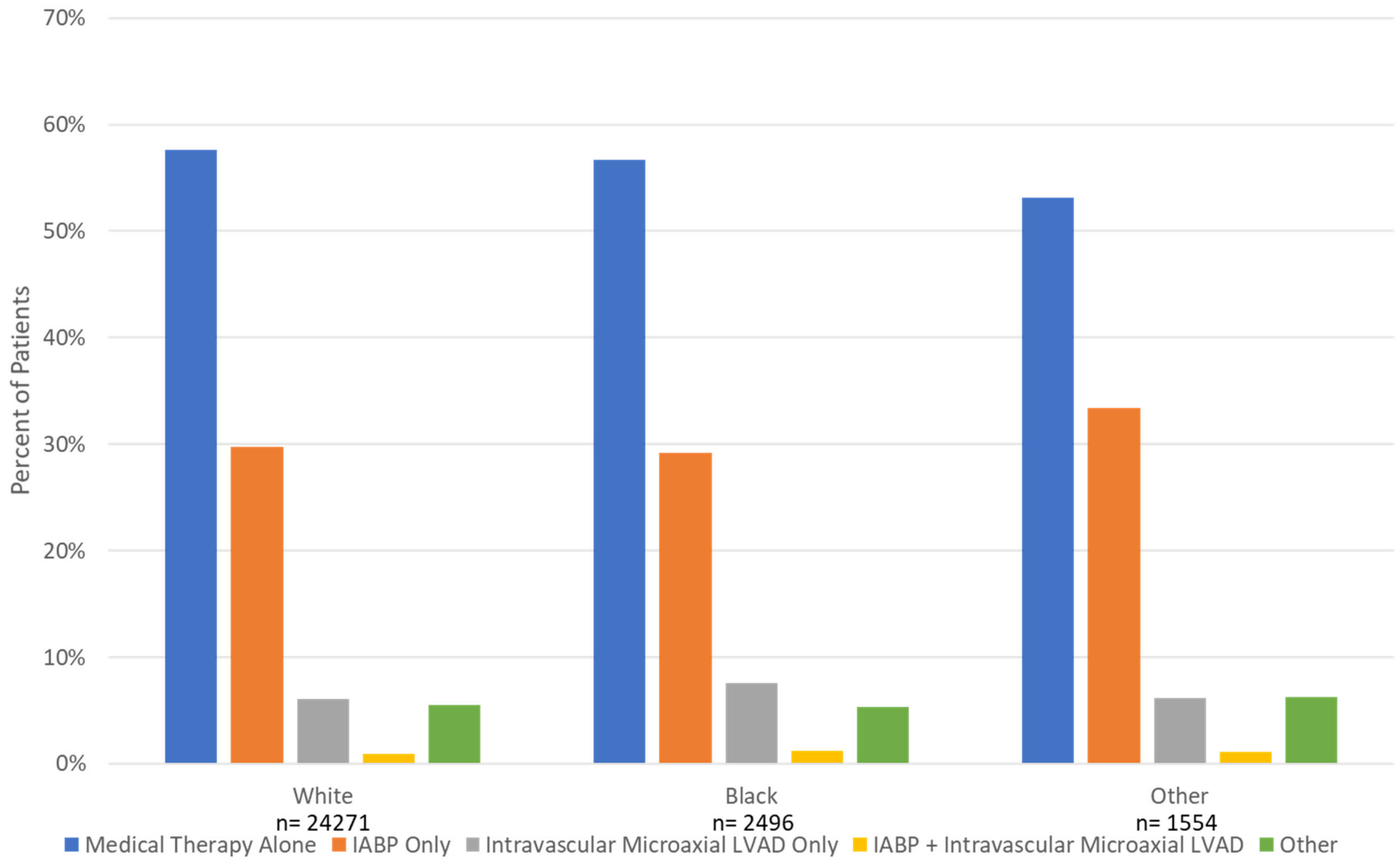

©2021 Dhruva SS et al. *JAMA Network Open.*

eFigure 5. Insurance Distribution by Therapy for Patients Undergoing Percutaneous Coronary Intervention for Acute Myocardial Infarction Complicated by Cardiogenic Shock from October 1, 2015 – December 31, 2017

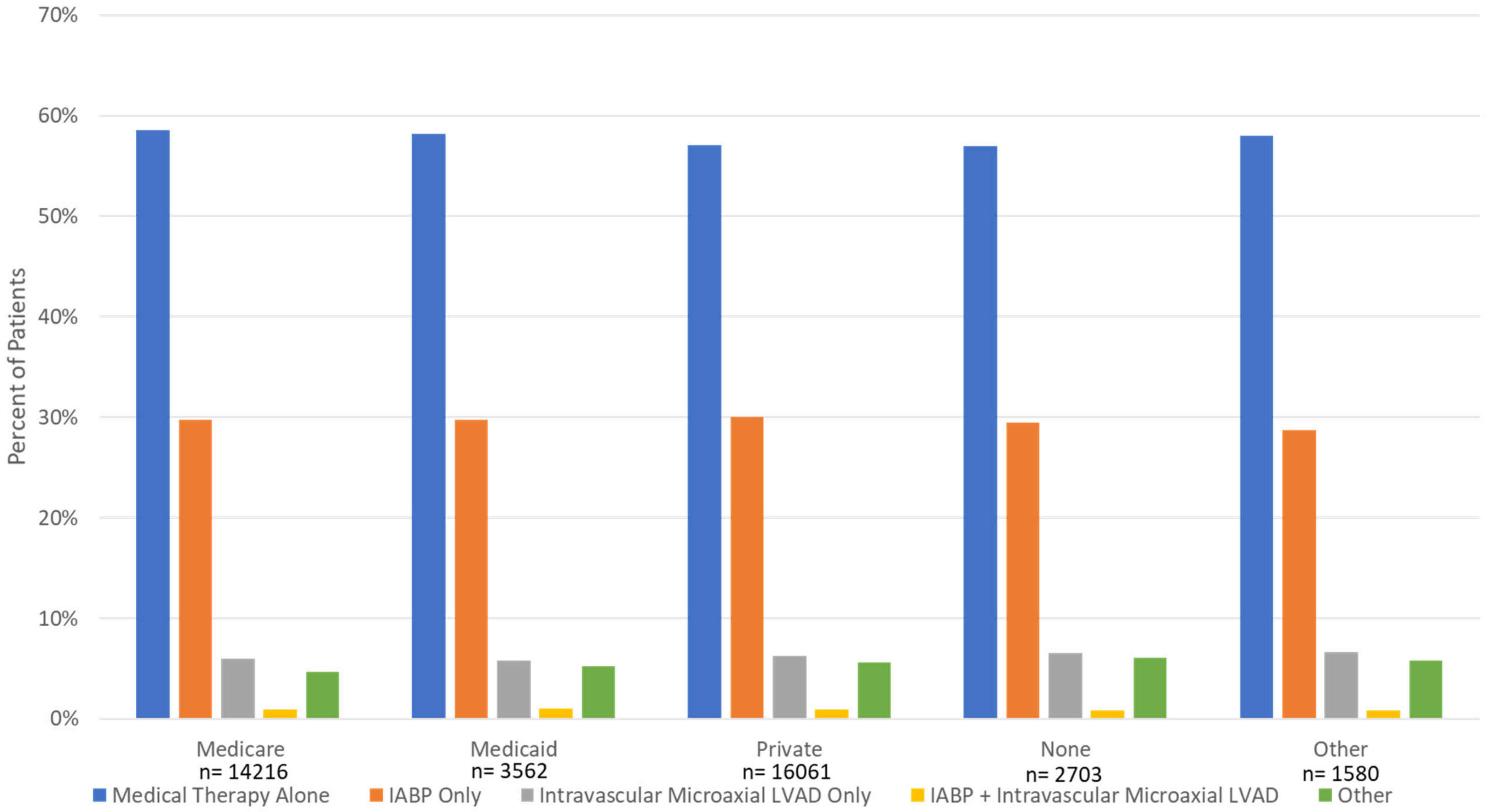

©2021 Dhruva SS et al. *JAMA Network Open.*

eFigure 6. Type of Myocardial Infarction by Therapy for Patients Undergoing Percutaneous Coronary Intervention for Acute Myocardial Infarction Complicated by Cardiogenic Shock from October 1, 2015 – December 31, 2017

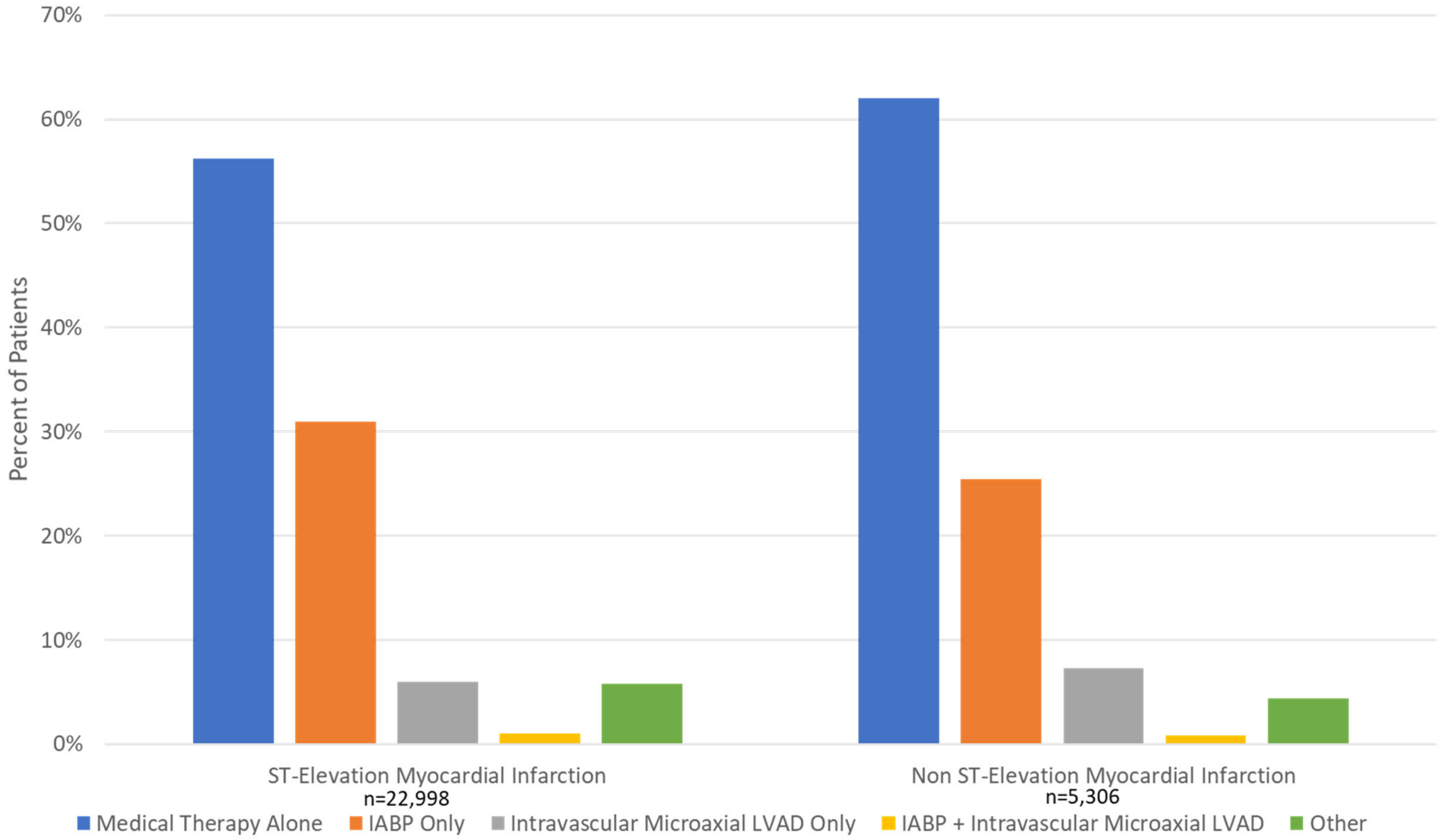

©2021 Dhruva SS et al. *JAMA Network Open.*

eFigure 7. Cardiac Arrest Status by Therapy for Patients Undergoing Percutaneous Coronary Intervention for Acute Myocardial Infarction Complicated by Cardiogenic Shock from October 1, 2015 – December 31, 2017

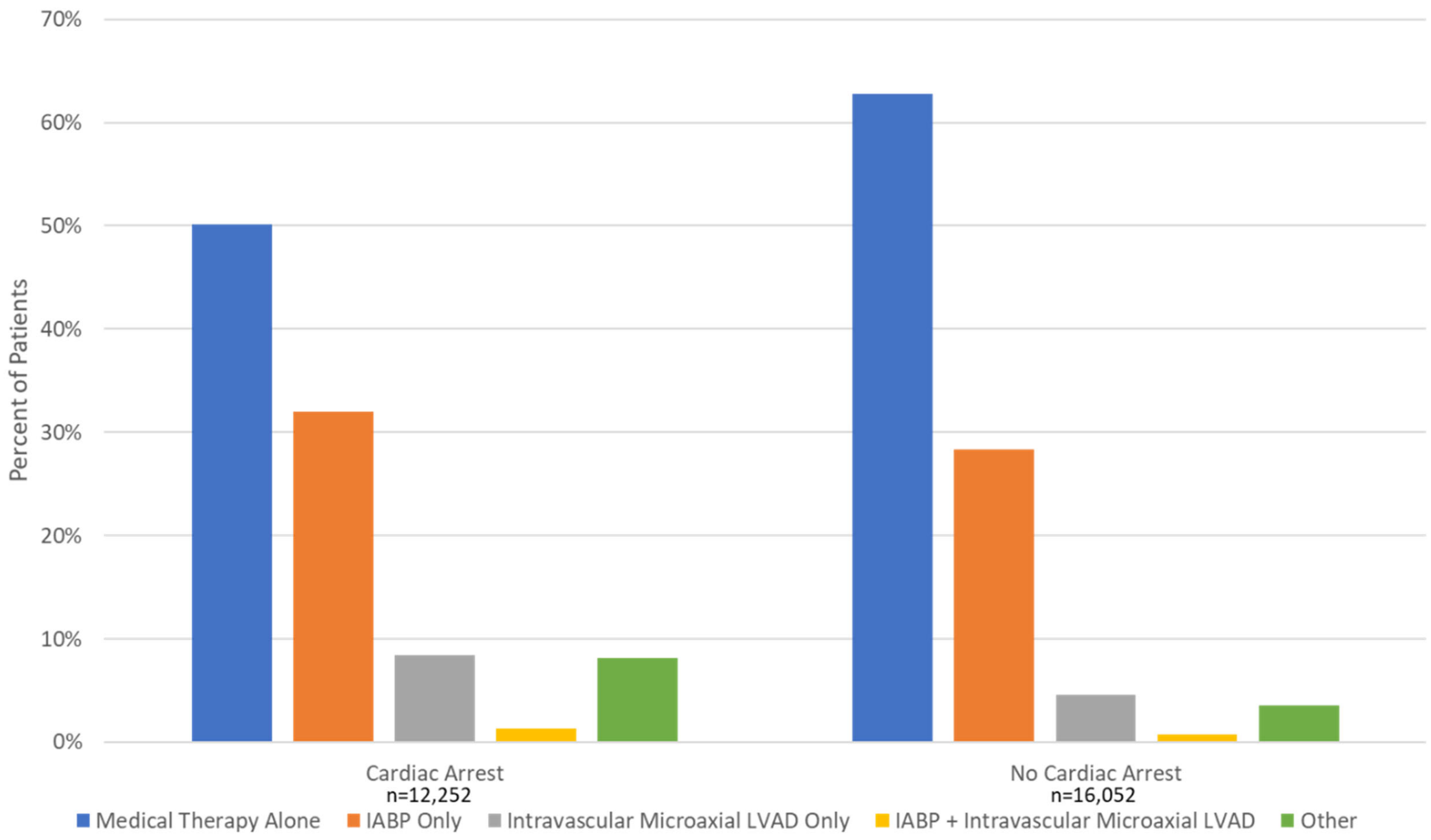

©2021 Dhruva SS et al. *JAMA Network Open.*

eFigure 8. Therapies for Transfer Patients Undergoing Percutaneous Coronary Intervention for Acute Myocardial Infarction Complicated by Cardiogenic Shock from October 1, 2015 – December 31, 2017

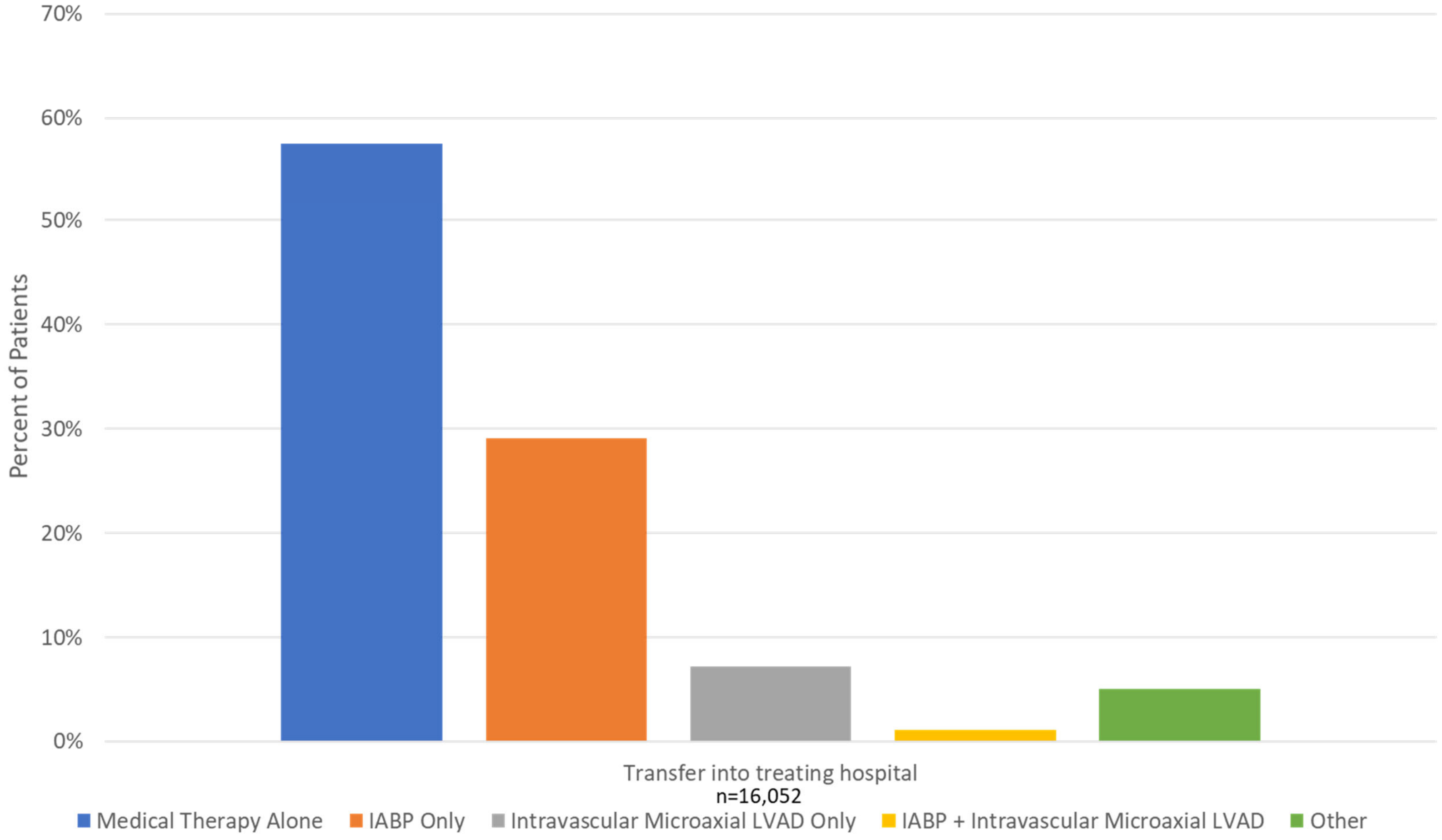

©2021 Dhruva SS et al. *JAMA Network Open.*
